# Relationship between left main and left anterior descending arteries bifurcation angle and coronary artery calcium score in chronic kidney disease: A 3-dimensional analysis of coronary computed tomography

**DOI:** 10.1371/journal.pone.0198566

**Published:** 2018-06-12

**Authors:** Takao Konishi, Naohiro Funayama, Tadashi Yamamoto, Daisuke Hotta, Shinya Tanaka

**Affiliations:** 1 Department of Cardiology, Hokkaido Cardiovascular Hospital, Sapporo, Japan; 2 Department of Cancer Pathology, Hokkaido University, Graduate School of Medicine, Sapporo, Japan; Michigan State University, UNITED STATES

## Abstract

**Background:**

A high coronary artery calcium score (CACS) predicts a poor prognosis in patients with coronary artery disease. We examined the relationship between the bifurcation angle and the CACS of the left main (LM) and left anterior descending (LAD) arteries in patients suffering from chronic kidney disease (CKD).

**Methods:**

We analyzed the data of 121 patients who underwent coronary computed tomography between October 2014 and June 2015 and whose estimated glomerular filtration rate (eGFR) was <60 ml/min/1.73 m^2^. The LM-LAD bifurcation angle was measured by 3-dimensional coronary computed tomography. The CACS of the LM-LAD arteries was also calculated. We excluded stent recipients and patient who had undergone coronary artery bypass graft surgery.

**Results:**

In the overall sample, the mean ± standard deviation (range) LM-LAD bifurcation angle was 35.9 ± 11.4° (6.8–79.4°) and mean CACS was 227 ± 351 (0 to 1,695). The mean LM-LAD arteries angle was 40.3° ± 10.0° in 39 patients whose CACS was ≥200, versus 33.8° ± 11.6° in 82 patients with CACS <200 (*p* = 0.003). A weak, but positive correlation (r = 0.269, *p* = 0.003) was observed between the LM-LAD arteries angle and CACS of the LM-LAD arteries. By multiple variable analysis, hemoglobin A1c, triglycerides, eGFR and the LM-LAD arteries angle were independent predictors of a high CACS of the LM-LAD arteries.

**Conclusion:**

In patients with CKD, a wide LM-LAD arteries angle was associated with a high CACS of the LM-LAD arteries. The prognostic value of this observation warrants further evaluation.

## Introduction

In the past three decades, computed tomography angiography (CTA) has been adopted for its high spatial and temporal resolution and accuracy as a superior imaging test for the diagnosis of coronary artery disease [1–4]. Coronary CTA enables the visualization of coronary artery anatomy as well as the composition of coronary atherosclerotic plaques. The assessment of the left coronary bifurcation angle has gained clinical interest because the angulation of the left coronary bifurcation has been shown to influence the wall shear stress and cause disturbances of bloodstream followed by the atherosclerotic development at the bifurcation region [5–7]. Therefore, analyses of the bifurcation angles will provide clinically important information about the relationship between coronary atherosclerosis and coronary bifurcation angles. In previous studies, measurement of the left coronary bifurcation angle has been found useful to predict high-grade coronary stenoses [5, 6] and clinical outcomes after percutaneous coronary intervention in the left coronary artery [8–10]. Since the left anterior descending (LAD) coronary artery supplies approximately 50% of the left ventricular myocardial blood flow [11, 12], progression of atherosclerosis in that vessel might be associated with worse clinical outcomes than progression of disease in other epicardial arteries [13].

Coronary artery calcium score (CACS), a measurement of subclinical coronary atherosclerosis, is a strong, independent predictor of coronary artery disease and cardiovascular events [14–16]. The contributions of CACS measurements have also been studied in patients with chronic kidney disease (CKD), in whom coronary calcifications are more prevalent, widespread, severe and progressive than in patients without CKD [17, 18].

Although some reports have demonstrated that angle between LAD artery and left circumflex (LCX) artery (LAD-LCX arteries angle) is a predictor of significant coronary atherosclerotic plaques in LAD or LCX artery [5, 6], no study has directly examined whether LM-LAD arteries angle and the development of LAD artery calcifications are correlated in patients suffering from CKD. This study examined our hypothesis of a causal relationship between the characteristics of the LM-LAD arteries angle and the CACS of LM-LAD arteries in patients suffering from CKD.

## Study sample and methods

### Data collection and follow-up

We screened the data from 787 consecutive patients who had undergone coronary CTA between October 2014 and June 2015 at Hokkaido Cardiovascular Hospital, Japan. We excluded 165 patients who had undergone a previous percutaneous coronary intervention or coronary artery bypass graft surgery at the time of CTA and 501 patients whose estimated glomerular filtration rate (eGFR) was ≥60 ml/min^–1^/1.73 m^2^ for ≥3 months. The 121 eligible patients were followed for three years after coronary CTA. Major adverse cardiovascular event (MACE) was defined as cardiac death, Q-wave myocardial infarction and any surgical or percutaneous coronary revascularization. Major adverse limb event (MALE) included acute limb ischemia, any peripheral revascularization and major amputations [19]. This study was approved by the research ethics committee at Hokkaido Cardiovascular Hospital and complies with the Declaration of Helsinki. Informed consent to participate was obtained from the patients or from a relative prior to coronary CTA.

### Coronary CTA protocol

Retrospective ECG-gated coronary CTA was performed on a 64-detector Ingenuity Core 64 CTA scanner (Philips, Cleveland, USA) with a gantry rotation of 0.35 s and a collimation of 40 mm. Patients with heart rates ≥70 bpm received oral landiolol or propranolol and sublingual nitroglycerin before the scan. Test injection technique was used for all coronary CTA scans. The scan was performed with a 260-mgI/kg intravenous injection of iopamidol (Iopamiron® 370 mgI/ml; Bayer, Osaka, Japan) at a flow rate of 26 mgI/kg/sec, followed by a 30-ml saline flush at the same flow rate. The images were acquired between 2 cm above the level of the LM artery and 2 cm below the cardiac apex. The images were routinely reconstructed at 40±5% and 75±10% of the R-R interval, with a slice thickness of 0.625 mm. The CACS was calculated by multiplying the area of each calcified lesion by a weighting factor corresponding to the peak pixel intensity of each lesion [20]. The calcium threshold was set at 130 Hounsfield units. Total CACS, CACS of the LM + LAD arteries, LM + LCX and CACS of the right coronary (RCA) artery were used in the analysis. The radiation doses ranged between 0.7 and 2 mSv.

In previous studies, A) an Agatston score ≥400 was considered indicative of severe calcifications and was a predictor of high-grade coronary stenoses and adverse clinical outcomes independently of other cardiovascular risk factors [16, 21], and B) the CACS of the LM-LAD arteries was 40–61% of total CACS [21–23]. Therefore, we chose a cut-off value of 200 in our search of correlates of CACS of the LM-LAD arteries.

### Reconstruction and analysis of the CT images

**Fig 1:**
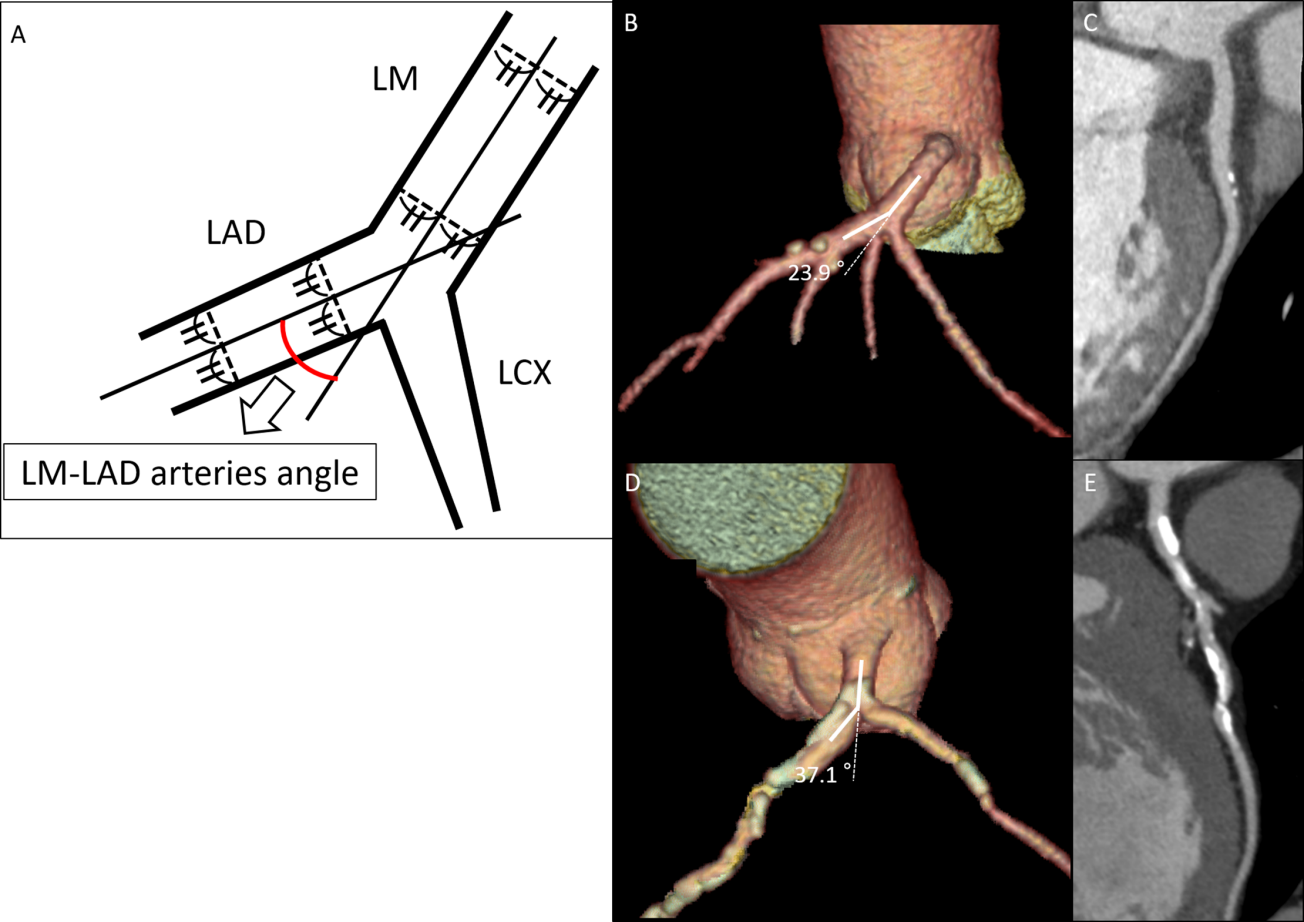
**A.** Schematic representation of the LM-LAD arteries angle measurements. We identified the lines that delimited the LM-LAD angle, using the centre lines of the LM and LAD arteries on the volume rendering image. **B, C.** Representative example of mild coronary calcifications in a 69-year-old man. The LM–LAD arteries angle measured 23.9° and the CACS in the LM-LAD arteries was 14.7. **D, E.** Representative example of severe coronary calcifications in a 63-year-old man. The LM–LAD arteries angle measured 37.1° and the CACS in the LM-LAD arteries was 783. LM = left main artery; LAD = left anterior descending artery; LCX = left circumflex artery.

All CT images were transferred to an Advantage Volume Share 4.6 workstation (GE Healthcare Japan Inc., Hino, Japan) for further analysis by two independent, highly experienced observers (with more than 11 years’ experience in cardiac CT imaging), unaware of the clinical status and identity of the patients. Three-dimensional volume rendering images reconstructed from a 2-dimensional axial coronary CTA were used to measure the LM-LAD angle. The LM-LAD arteries angle was measured three times in end-diastole and the measurements were averaged. Fig 1A is a schematic representation of the measurements of a LM-LAD angle, and Fig 1B, 1C and 1D, 1E are CTA images of mild and severe coronary calcifications, respectively. The inter-observer variability was evaluated by comparing the measurements made by the two independent observers, who used identical methods.

### Statistical analysis

The data are expressed as means ± standard deviation. Between-group differences were examined, using the Pearson chi-square test or Fisher exact test for categorical variables and the Student *t*-test or Mann–Whitney U-test for continuous variables, as appropriate. The relationship between LM-LAD arteries angle and CACS was determined with Pearson’s correlation. Receiver-operating characteristic curves were constructed to identify the optimal predictor of high CACS of LM-LAD artery. To identify independent predictors of high CACS of LM-LAD arteries, a multiple variable logistic regression analysis, which included variables with *p* <0.05, was performed for each parameter used as the dependent variable. Odd ratios and 95% confidence intervals were calculated to ascertain the significance of the differences. A *p* value <0.05 was considered statistically significant. All analyses were performed using the JMP version 13 (SAS Institute, Cary, NC, USA).

## Results

### Clinical observations

The mean age of the 121 eligible patients enrolled in this study was 73.7 ± 8.3 years, 64% were men, 26% were diabetic and 78% were hypertensive. Their mean eGFR was 46.5 ± 15.2 ml/min/1.73 m^2^. The mean LM-LAD arteries angle was 35.9 ± 11.4° (range 6.8° to 79.4°). The mean CACS of the LM-LAD arteries was 227 ± 351 (range 0 to 1,695).

Table 1 shows the clinical characteristics of the study groups. The mean LM-LAD arteries angle in 39 patients whose CACS was ≥200 (40.3° ± 10.0°) was significantly wider (*p* = 0.003) than in 82 patients whose CACS was <200 (33.8° ± 11.6°). The prevalence of dyslipidemia and hemodialysis and mean hemoglobin A1c were significantly higher, and the mean high-density lipoprotein (HDL) cholesterol and mean estimated glomerular filtration rate (eGFR) were significantly lower in the group whose CACS was ≥200 than in the group whose CACS was <200. All other characteristics were similar in both groups (Table 1).

**Table 1:** Baseline characteristics of 82 patients with <200 versus 39 patients with ≥200 coronary artery calcium score (CACS) of the left main (LM)-left anterior descending (LAD) arteries.

	CACS of LM-LAD arteries		*p*
	<200 (n = 82)	≥200 (n = 39)	
Age (years)	74.8±7.9	71.5±8.9	0.053
Men	49 (60)	29 (74)	0.117
Body mass index	24.0±4.0	23.8±3.6	0.797
History of:			
Diabetes mellitus	17 (21)	14 (36)	0.074
Hypertension	64 (78)	30 (77)	0.889
Dyslipidemia	55 (67)	34 (87)	0.019
Hemodialysis	4 (5)	8 (21)	0.018
Current smoking	17 (21)	10 (26)	0.544
Sleep apnea syndrome	8 (10)	4 (10)	0.811
Transient ischemic attack or stroke	12 (15)	6 (15)	0.914
Peripheral artery disease	7 (9)	9 (23)	0.055
Family history of coronary artery disease	7 (9)	1 (3)	0.399
History of drug therapy			
Statin	30 (37)	17 (44)	0.460
Aspirin	16 (20)	11 (28)	0.283
Clopidogrel	5 (6)	5 (13)	0.367
Cilostazol	2 (2)	3 (8)	0.175
Ticlopidine	2 (2)	1 (3)	0.967
Angiotensin converting enzyme inhibitor or receptor blocker	38 (46)	17 (44)	0.776
Calcium channel blocker	39 (48)	19 (49)	0.905
Beta-adrenergic blocker	23 (28)	11 (28)	0.986
Vitamin D	4 (5)	4 (10)	0.266
Calcium carbonate	3 (4)	6 (15)	0.054
Warfarin	11 (13)	7 (18)	0.512
Blood pressure, mmHg			
Systolic	135±18	135±20	0.899
Diastolic	77±14	77±11	0.981
Hemoglobin, g/dl	13.3±1.7	13.1±2.0	0.575
Hemoglobin A1c, %	5.8±0.5	6.1±0.8	0.025
Glucose, mg/dl	113±37	122±42	0.214
Cholesterol, mg/dl			
Low-density lipoprotein	106±30	109±41	0.715
High-density lipoprotein	56±16	49±15	0.026
Low-density/high-density lipoprotein cholesterol ratio	2.1±0.9	2.4±1.1	0.150
Triglyceride, mg/dl	143±88	161±92	0.324
Estimated glomerular filtration rate, ml/min/1.73m^2^	48.9±12.2	41.6±19.6	0.036
Corrected serum calcium, mg/dl	9.2±0.4	9.1±0.5	0.436
Degree of left main-left anterior descending coronary arteries angle	33.8±11.6	40.3±10.0	0.003

Values are means ± standard deviations or numbers (%) of observations

**Fig 2:**
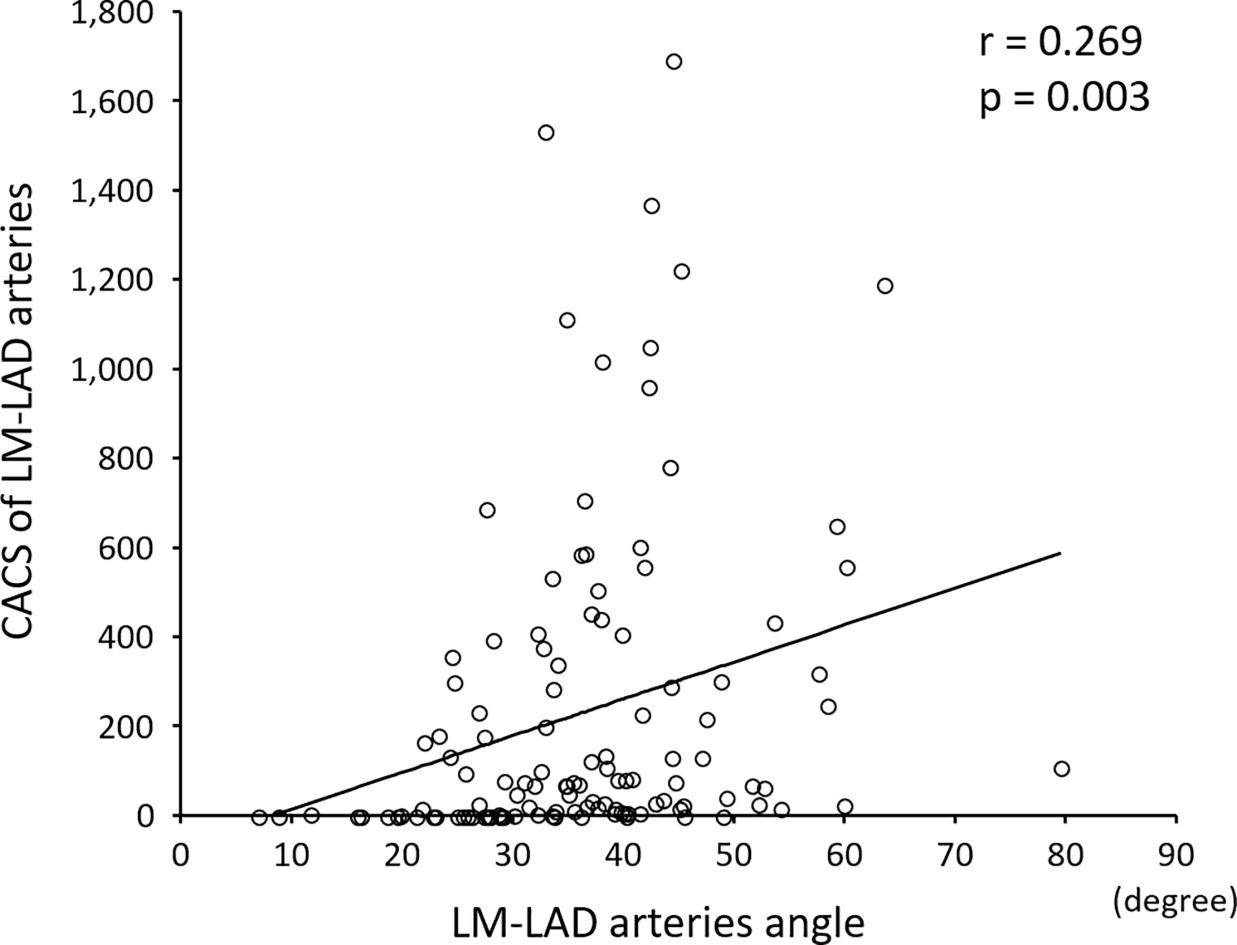
A weak positive correlation between the LM-LAD arteries angle and the CACS of the LM-LAD arteries (r = 0.269; *p* = 0.003).

A weak, but positive correlation (r = 0.269, *p* = 0.003) was observed between the LM-LAD arteries angle and CACS of the LM-LAD arteries (Fig 2). The LM-LAD arteries angle was slightly correlated with total CACS (r = 0.232; *p* = 0.010; S1A Fig) and with CACS of the LM-LCX arteries (r = 0.185; *p* = 0.042; S1B Fig). However, there was no correlation between the LM-LAD arteries angle and CACS of the RCA artery (r = 0.158, *p* = 0.084; S1C Fig).

**Fig 3:**
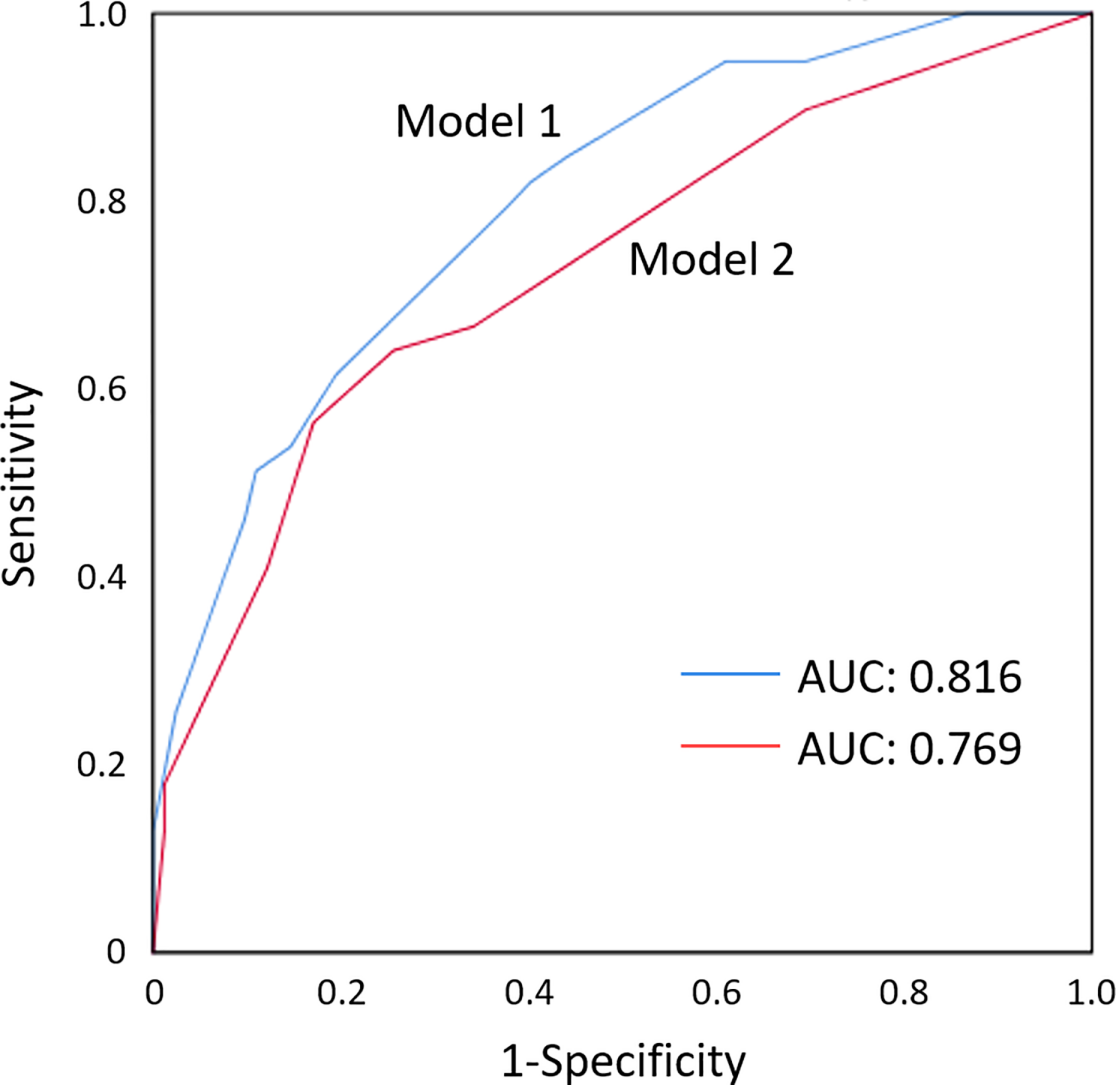
Comparison of diagnostic characteristics in model 1 (hemoglobin A1c, triglyceride, eGFR, LM-LAD arteries angle) versus model 2 (hemoglobin A1c, triglyceride, eGFR) to predict severe calcifications of the LM-LAD arteries. The accuracy of the C statistics in the prediction of a high CACS in the LM-LAD arteries was increased by adding the measurement of the LM-LAD arteries angle (area under the curve = 0.816 in model 1 versus 0.769 in model 2).

By receiver-operating characteristic (ROC), the optimal cut-off value of the LM-LAD arteries angle that predicted a high CACS of the LM-LAD arteries, was 32.1°, with a sensitivity, specificity, positive and negative predictive values, and a diagnostic accuracy of 87, 46, 44, 88 and 60%, respectively. Single and multiple variable logistic regression analyses were performed to identify independent predictors of high CACS of the LM-LAD arteries (Table 2). Patient age, dyslipidemia, hemodialysis, hemoglobin A1c, triglyceride, HDL-cholesterol, LDL-/HDL-cholesterol ratio, eGFR and LM-LAD arteries angle were included in the multiple variable model. We excluded dyslipidemia from the multiple variable analysis when we entered triglyceride, HDL-cholesterol, LDL-/HDL-cholesterol ratio in the analysis because they were mutually correlated. We similarly excluded hemodialysis when we entered eGFR in the multiple variable model. By multiple variable analysis, hemoglobin A1c, triglyceride, eGFR and the LM-LAD arteries angle were independent predictors of a high CACS of the LM-LAD arteries (Model 1 in Table 2). A wide LM-LAD arteries angle was associated with an increased risk of high CACS of the LM-LAD arteries (OR = 4.82; 95% CI 1.68–16.1; *p* = 0.003). Other multiple variable analysis models yielded similar results (S1 Table). The accuracy of the C statistics for the prediction of a high CACS of the LM-LAD arteries was significantly increased by the addition of the LM-LAD arteries angle (area under the curve: 0.816; model 1 in Fig 3) to the model, including other risk factors (area under the curve: 0.769; model 2 in Fig 3). The correlation of the LM-LAD angles measured by the two independent observers was r = 0.908; *p* <0.001.

**Table 2:** Outcomes of single and multiple variable logistic regression analyses of correlates of CACS of the LM-LAD arteries <200 versus ≥200.

	ANALYSIS			
	Single variable		Multiple variable	
	Odd ratio(95% CI)	*p*	Odd ratio(95% CI)	*p*
Age (years)	0.25 (0.10–0.63)	0.005	0.44 (0.14–1.37)	0.153
Men	1.95 (0.84–4.54)	0.172		
Body mass index	0.43 (0.15–1.24)	0.173		
History of:				
Diabetes mellitus	2.14 (0.92–4.98)	0.118		
Hypertension	0.94 (0.38–2.33)	0.925		
Dyslipidemia	3.34 (1.17–9.50)	0.033		
Hemodialysis	5.03 (1.41–17.9)	0.018		
Current smoking	1.32 (0.54–3.23)	0.709		
Sleep apnea syndrome	1.06 (0.30–3.75)	0.811		
Transient ischemic attack or stroke	1.06 (0.37–3.07)	0.869		
Peripheral artery disease	3.21 (1.10–9.41)	0.055		
Family history of coronary artery disease	0.28 (0.03–2.38)	0.399		
History of drug therapy				
Statin	1.34 (0.62–2.91)	0.590		
Aspirin	1.62 (0.67–3.93)	0.401		
Clopidogrel	2.26 (0.61–8.34)	0.367		
Cilostazol	3.33 (0.53–20.8)	0.385		
Ticlopidine	1.05 (0.09–12.0)	0.559		
Angiotensin converting enzyme inhibitor or receptor blocker	0.89 (0.42–1.93)	0.929		
Calcium channel blocker	1.05 (0.49–2.25)	0.940		
Beta-adrenergic blocker	1.01 (0.43–2.35)	0.843		
Vitamin D	2.23 (0.53–9.43)	0.471		
Calcium carbonate	4.79 (1.13–20.3)	0.054		
Warfarin	1.41 (0.50–3.98)	0.703		
Blood pressure, mmHg				
Systolic	1.59 (0.61–4.13)	0.477		
Diastolic	0.32 (0.07–1.48)	0.221		
Hemoglobin, g/dl	0.42 (0.17–1.04)	0.098		
Hemoglobin A1c, %	3.63 (1.59–8.28)	0.003	3.21 (1.25–8.46)	0.015
Glucose, mg/dl	2.30 (1.04–5.05)	0.059		
Cholesterol, mg/dl				
Low-density lipoprotein	1.92 (0.89–4.16)	0.141		
High-density lipoprotein	0.30 (0.12–0.73)	0.011	0.61 (0.21–1.69)	0.344
Low-density/high-density lipoprotein cholesterol ratio	2.50 (1.14–5.45)	0.033		
Triglyceride, mg/dl	2.63 (1.14–6.09)	0.036	2.78 (1.06–7.86)	0.038
Estimated glomerular filtration rate, ml/min/1.73m^2^	0.31 (0.12–0.75)	0.015	0.29 (0.09–0.88)	0.029
Corrected serum calcium, mg/dl	0.45 (0.20–1.00)	0.075		
Degree of left main-left anterior descending coronary arteries angle	5.87 (2.09–16.5)	<0.001	4.82 (1.68–16.1)	0.030

Over the three-year follow-up, 18 of the 39 patients (46.2%) with CACS of the LM-LAD arteries ≥200 suffered MACE or MALE versus 10 of the 82 patients (12.2%) with CACS <200 (*p* <0.001; S2 Table). Furthermore, over the three-year follow-up, 21 of the 61 patients (34.4%) with LM-LAD arteries angle ≥35.8˚, which is the optimal cut-off for the prediction of MACE or MALE, suffered these two events, versus 7 of 60 patients (11.6%) with LM-LAD arteries angle <35.8˚ (*p* = 0.003; S3 Table).

## Discussion

The main observations made in this study are 1) hemoglobin A1c, triglycerides, eGFR and the LM-LAD arteries angle were predictors of high CACS in patients suffering from CKD, and 2) in these patients, the CACS was higher when the LM-LAD arteries angle was ≥ than when it was <32.1°. To the best of our knowledge, this study is the first to a) find a correlation between the LM-LAD arteries angle and CACS in patients suffering from CKD, and b) compare the LM-LAD arteries angle with other predictors of high CACS by multiple variable analysis. It adds important anatomical information to the list of predictors of severe coronary calcifications. Therefore, its merit, as a predictor of clinical cardiovascular events in patients suffering from CKD warrant further evaluation.

### Comparison with previous measurements of angulation

Our mean measurement of the LM-LAD arteries angle was 35.9 ± 11.4°, closely concordant with the average bifurcation angles measured in previous studies. Using coronary CTA, the mean LM-LAD arteries angle measured by Kawasaki et al. was 37±13° [24], and that by Cui et al. was 34.2±13.4° [6]. In a previous analysis of coronary angiographies, we measured an average LM-LAD arteries angle of 34.1°±18.5° [9].

### LM-LAD angle, wall shear stress and atherosclerosis and calcifications

The left coronary bifurcation angle may influence the development and progression of atherosclerosis [5–7] and the clinical outcome after percutaneous coronary intervention for left coronary artery disease [8–10]. Several studies have observed that widely angulated left coronary artery bifurcations are associated with low wall shear stress gradient caused by disturbances of the bloodstream, which promotes atherosclerotic progression at the site of bifurcation [25]. In an intravascular ultrasound study, coronary calcifications was positively correlated with the atherosclerotic plaque burden [26]. Therefore, a wide LM-LAD arteries angle in patients suffering from CKD might lead to plaque progression, resulting in a high CACS in this study. The use of a cut-off value of 32.1° for the LM-LAD arteries angle in our multiple variable analysis preserved the statistical significance after adjustments for confounding factors, suggesting that the LM-LAD arteries angle is a reliable predictor of CACS of the LM-LAD artery (Table 2, S1 Table).

According to ROC analysis, the diagnostic performance of the LM-LAD arteries angle showed comparatively low specificity and positive predictive value (PPV) of 46 and 44% for predicting high CACS of the LM-LAD arteries. We speculate that this is because coronary bifurcation angle could become a significant predictor of coronary artery calcification only after being added other risk factors such as diabetes, dyslipidemia and CKD. When a study included only hemodialysis patients, the specificity and PPV might be higher and false positive rates would be lower than the current study.

### Mechanisms of calcifications in patients suffering from CKD

Autopsies of patients suffering from CKD have revealed a high prevalence of vascular wall calcifications [27, 28], which are a reliable marker of coronary atherosclerotic plaque burden. Fibroblast growth factor 23, which is increased during the early stages of CKD, and serum Klotho, which is decreased in patients with CKD, are both associated with vascular calcifications [29, 30]. In an epidemiologic study, high concentrations of fibroblast growth factor 23 were associated with a risk of developing cardiovascular atherosclerosis [31]. Furthermore, recent studies have reported a relationship between low serum Klotho concentrations in patients suffering from CKD and the presence and severity of cardiovascular atherosclerosis, independently of known cardiovascular risk factors [32].

### Other observations

In this study, increased triglyceride and HbA1c is associated with high CACS of LM-LAD arteries (Table 2). Increased triglyceride is associated with valvular and vascular calcification [33, 34]. Diabetes mellitus (DM) is one of well-known risk factors of coronary calcification [35, 36], and even in patients without DM, HbA1c is independently associated with coronary artery calcification [37]. The results in our study is well concordant with these previous reports.

### Limitations of our study

The sample size of this retrospective, observational, single-centre study was small. Its results need to be confirmed prospectively in a larger study. Second, we did not measure other factors, for example serum fibroblast growth factor 23 and Klotho, which may be associated with vascular calcifications in patients suffering from CKD. Third, since our data collection was based on a review of medical records, we did not gather detailed clinical data over the three-year follow-up.

### Clinical implications

Despite these limitations, our study showed that a wide LM-LAD arteries angle is one of the several factors associated with severe calcifications of these arteries. The LM-LAD arteries angle provides clinically important information toward the prediction of adverse clinical events, since wide angles are associated with high CACS, which should prompt the attentive management of all major risk factors, with a view to optimise the clinical outcomes in patients suffering from CKD.

## Conclusion

The results of this study suggest that, in patients suffering from CKD, a wide LM-LAD arteries angle is associated with a high CACS of the bifurcation. Measurement of that angle should be part of clinical practice to identify patients suffering from CKD at high risk of developing severe calcifications, which might burden their prognosis.

## Supporting information

S1 Fig **Relationship between LM-LAD arteries angle and A. total, B. LM-LCX, and C. RCA CACS.** The LM-LAD arteries angle was slightly correlated with total CACS (r = 0.232, *p* = 0.010) and CACS of the LM-LCX arteries (r = 0.185, *p* = 0.042), but not with CACS of RCA (r = 0.158, *p* = 0.084).
(TIF)

S1 Table Multiple variable analysis in models 2–6. (DOCX)

S2 Table Incidence of MACE and MALE in relationship to the CACS of the LM-LAD arteries. (DOCX)

S3 Table Incidence of MACE and MALE in relationship to the LM-LAD arteries angle. (DOCX)
