# Supplementary figures and images for: Relationship between left main and left anterior descending arteries bifurcation angle and coronary artery calcium score in chronic kidney disease: A 3-dimensional analysis of coronary computed tomography
Source: PLoS One. 2018 Jun 12;13(6):e0198566. doi: 10.1371/journal.pone.0198566 (PMC5997324; doi:10.1371/journal.pone.0198566)

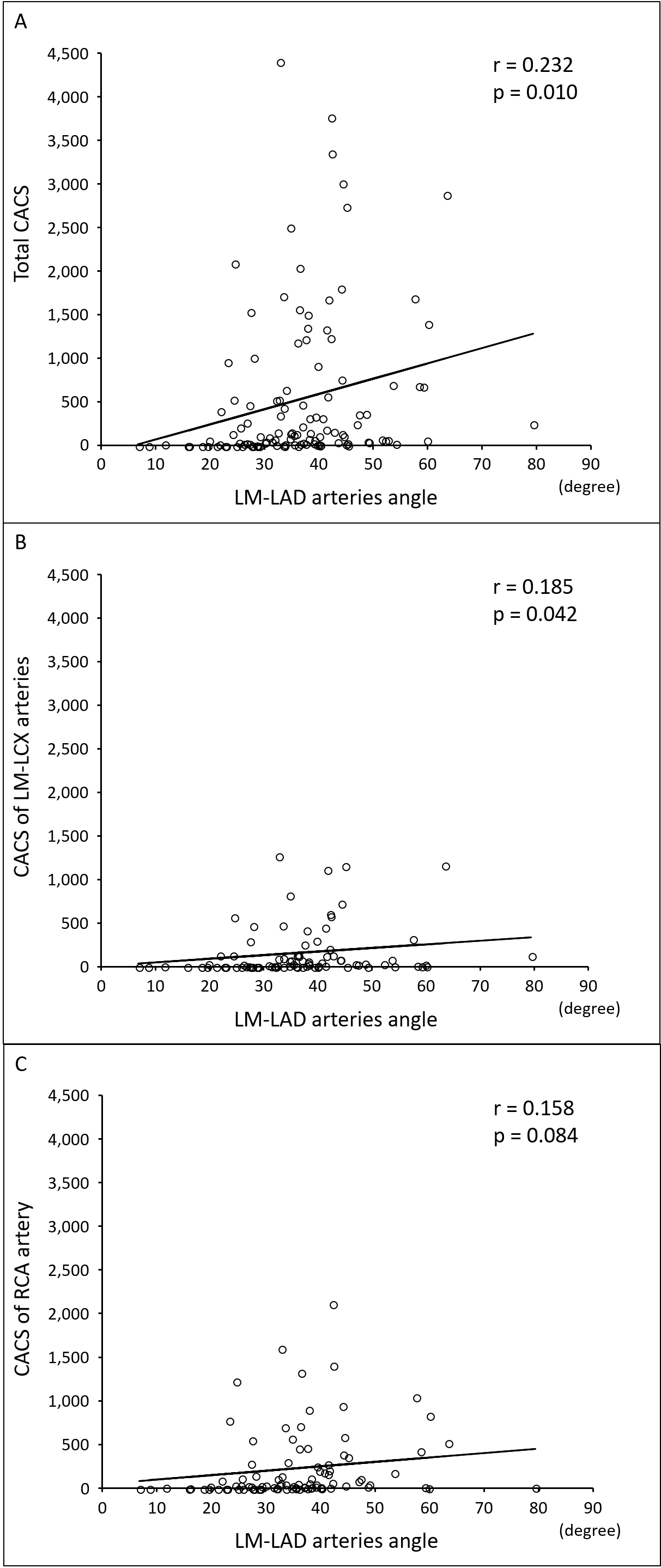

Supplement: S1 Fig — Relationship between LM-LAD arteries angle and A. total, B. LM-LCX, and C. RCA CACS. The LM-LAD arteries angle was slightly correlated with total CACS (r = 0.232, p = 0.010) and CACS of the LM-LCX arteries (r = 0.185, p = 0.042), but not with CACS of RCA (r = 0.158, p = 0.084). (TIF) [file pone.0198566.s001.tif]
