# Supplementary material for: Relationship between left main and left anterior descending arteries bifurcation angle and coronary artery calcium score in chronic kidney disease: A 3-dimensional analysis of coronary computed tomography
Source: PLoS One. 2018 Jun 12;13(6):e0198566. doi: 10.1371/journal.pone.0198566 (PMC5997324; doi:10.1371/journal.pone.0198566)
Supplement: S1 Table — (DOCX) [file pone.0198566.s002.docx]

**S1 Table. Multiple variable analysis in models 2 - 6**

|  | **MODELS** | | | | | | | | | |
| --- | --- | --- | --- | --- | --- | --- | --- | --- | --- | --- |
|  | **2** | | **3** | | **4** | | **5** | | **6** | |
| **Variables** | **OR (95% CI)** | ***p*** | **OR (95% CI)** | ***p*** | **OR (95% CI)** | ***P*** | **OR (95% CI)** | ***p*** | **OR (95% CI)** | ***p*** |
| Age, y | 0.42 (0.14-1.30) | 0.132 | 0.42 (0.14-1.30) | 0.132 | 0.49 (0.14-1.69) | 0.258 | 0.48 (0.14-1.64) | 0.241 | 0.49 (0.14-1.66) | 0.249 |
| Dyslipidemia |  |  | 3.55 (1.02-12.4) | 0.047 | 4.34 (1.20-15.6) | 0.025 |  |  |  |  |
| Hemodialysis |  |  |  |  | 3.92 (0.64-24.0) | 0.141 | 3.01 (0.51-17.6) | 0.223 | 3.26 (0.53-20.0) | 0.201 |
| Hemoglobin A1c, % | 3.86 (1.42-10.5) | 0.008 | 3.48 (1.36-8.95) | 0.010 | 3.24 (1.28-8.20) | 0.013 | 2.97 (1.17-7.55) | 0.022 | 3.75 (1.39-10.1) | 0.009 |
| Triglycerides, mg/dl | 2.74 (1.02-7.33) | 0.045 |  |  |  |  | 2.85 (1.06-7.68) | 0.038 | 2.84 (1.06-7.60) | 0.037 |
| HDL cholesterol, mg/dl |  |  |  |  |  |  | 0.54 (0.20-1.49) | 0.235 |  |  |
| LDL to HDL cholesterol ratio | 2.16 (0.83-5.64) | 0.116 |  |  |  |  |  |  | 2.54 (0.99-6.52) | 0.053 |
| Estimated GFR, ml/min/1.73 m^2^ | 0.32 (0.10-0.96) | 0.043 | 0.29 (0.10-0.85) | 0.025 |  |  |  |  |  |  |
| Left main-left anterior descending arteries angle, _˚_ | 5.08 (1.67-15.5) | 0.004 | 4.97 (1.64-15.1) | 0.005 | 3.83 (1.35-10.9) | 0.012 | 3.81 (1.32-11.0) | 0.013 | 4.06 (1.40-11.8) | 0.010 |

OR = odd ratio; CI = confidence interval; GFR = glomerular filtration rate; HDL = high-density lipoprotein; LDL = low-density lipoprotein
