# Supplementary material for: Relationship between left main and left anterior descending arteries bifurcation angle and coronary artery calcium score in chronic kidney disease: A 3-dimensional analysis of coronary computed tomography
Source: PLoS One. 2018 Jun 12;13(6):e0198566. doi: 10.1371/journal.pone.0198566 (PMC5997324; doi:10.1371/journal.pone.0198566)
Supplement: S2 Table — (DOCX) [file pone.0198566.s003.docx]

**S2 Table. Incidence of major adverse cardiovascular and limb events in relationship to the coronary artery calcifications score (CACS) of the left main (LM)-left anterior descending (LAD) arteries**

|  | **CACS of LM-LAD arteries** | | ***p*** |
| --- | --- | --- | --- |
|  | **<200 (n=82)** | **≥200 (n=39)** |  |
| **Major adverse cardiovascular events** |  |  |  |
| Cardiac death | 0 | 0 | - |
| Q-wave myocardial infarction | 0 | 0 | - |
| Surgical or percutaneous revascularisation | 9 (11.0) | 16 (41.0) | <0.001 |
| All adverse cardiovascular events | 9 (11.0) | 16 (41.0) | <0.001 |
| **Major adverse limb events** |  |  |  |
| Acute limb ischemia | 0 | 1 (2.6)* | 0.145 |
| Peripheral revascularization | 1 (1.2)* | 1 (2.6)* | 0.588 |
| Major amputation | 0 | 0 | - |
| All adverse limb events | 1 (1.2) | 2 (5.1) | 0.196 |
| **All adverse events** | 10 (12.2) | 18 (46.2) | <0.001 |

Values are numbers (%) of observations.

*One patient presenting with CACS of the LM-LAD arteries angle <200 and 2 patients with CACS ≥200
suffered major adverse cardiovascular and limb events within 3 years of follow-up.
