# Supplementary material for: Relationship between left main and left anterior descending arteries bifurcation angle and coronary artery calcium score in chronic kidney disease: A 3-dimensional analysis of coronary computed tomography
Source: PLoS One. 2018 Jun 12;13(6):e0198566. doi: 10.1371/journal.pone.0198566 (PMC5997324; doi:10.1371/journal.pone.0198566)
Supplement: S3 Table — (DOCX) [file pone.0198566.s004.docx]

**S3 Table. Incidence of major adverse cardiovascular and limb events**

**in relationship to the left main (LM)-left anterior descending (LAD) arteries angle**

|  | **LM-LAD arteries angle** | | ***p*** |
| --- | --- | --- | --- |
|  | **<35.8˚ (n=60)** | **≥35.8˚ (n=61)** |  |
| **Major adverse cardiovascular events** |  |  |  |
| Cardiac death | 0 | 0 | - |
| Q-wave myocardial infarction | 0 | 0 | - |
| Surgical or percutaneous revascularisation | 6 (10.0) | 19 (31.1) | 0.004 |
| All adverse cardiovascular events | 6 (10.0) | 19 (31.1) | 0.004 |
| **Major adverse limb events** |  |  |  |
| Acute limb ischemia | 0 | 1 (1.6)* | 0.319 |
| Peripheral revascularization | 1 (1.7)* | 1 (1.6)* | 0.991 |
| Major amputation | 0 | 0 | - |
| All adverse limb events | 1 (1.2) | 2 (3.3) | 0.569 |
| **All adverse events** | 7 (11.6) | 21 (34.4) | 0.003 |

Values are numbers (%) of observations.

*One patient presenting with CACS <200 and 2 patients with CACS ≥200 of the LM-LAD angle
suffered major adverse cardiovascular and limb events within 3 years of follow-up.
